# Clinical utility of the pan-immune-inflammation value in breast cancer patients

**DOI:** 10.3389/fonc.2023.1223786

**Published:** 2023-08-30

**Authors:** Xiaoyan Qi, Boyang Qiao, Tingting Song, Dan Huang, Hui Zhang, Yang Liu, Qi Jin, Ming Yang, Delong Liu

**Affiliations:** ^1^ Department of Breast Surgery, Liaoning Cancer Hospital & Institution, Shenyang, Liaoning, China; ^2^ Department of Breast Surgery, General Surgery Center, The First Hospital of Jilin University, Changchun, China; ^3^ School of Pharmaceutical Sciences, Wuhan University, Wuhan, China; ^4^ Department of Neurology and Neuroscience Center, The First Hospital of Jilin University, Changchun, China; ^5^ Department of Thoracic Surgery, Liaoning Cancer Hospital & Institution, Shenyang, Liaoning, China

**Keywords:** pan-immune-inflammation value, breast cancer, prognosis, clinical utility, meta-analysis

## Abstract

**Background:**

The newly discovered pan-immune-inflammation value (PIV) has been illustrated to have good prognostic value for cancer patient prognosis. However, the prognostic usefulness of PIV in breast cancer patients is unknown. As a result, to aid the clinic in providing a distinctive and trustworthy biomarker to better assess breast cancer patient’s prognosis, we conducted this meta-analysis to investigate the relationship between PIV and the survival of breast cancer patients.

**Methods:**

We conducted a systematic search of Pubmed, Embase, the Cochrane Library, and the CNKI databases to screen for eligible studies published up to April 2023. Outcomes included overall survival (OS), progression-free survival (PFS), and pathological complete response (pCR). The hazard ratio (HR) and the corresponding 95% confidence interval (CI) were used as the indicators. STATA 15.0 software was used to perform meta-analysis, sensitivity analysis, and publication bias analysis.

**Results:**

A total of eight articles, involving 2953 patients, met the inclusion criteria and were included in this meta-analysis. The results showed that patients with higher PIV levels had a significantly shorter OS (HR: 2.045, 95% CI: 1.355-3.086, *P* = 0.001) and PFS (HR: 1.466, 95% CI: 1.163-1.848, *P* = 0.001). Besides, the PIV value was negatively correlated with the efficacy of neoadjuvant chemotherapy. Sensitivity analysis showed that the results of this study were reliable and stable.

**Conclusions:**

PIV has a good prognostic value in breast cancer patients and is expected to be a prognostic biomarker for breast cancer.

## 1 Introduction

Globally, breast cancer has surpassed all other types of cancer in frequency among women (1, 2). 2.26 million new cases of breast cancer and 680,000 deaths among women worldwide were reported in 2020 (3). In addition, the incidence of breast cancer tends to increase year over year (4). Due to the ongoing breakthrough and success of systemic therapy, the mortality rate of breast cancer patients has decreased in recent years, and patient survival has increased (5, 6). Unfortunately, a subset of patients still has a poor prognosis. It is well understood that there is significant interindividual variation in treatment response, even among patients with breast cancer at the same clinical stage who receive the same treatment (7). Because of this prognostic heterogeneity (8, 9), it is necessary to identify appropriate prognostic parameters to better predict patient prognosis, personalize breast cancer treatment, and improve survival rates.

At present, many pieces of evidence have shown that the immune-inflammatory response of the host and the tumor interact in a complicated manner (10, 11). To a certain extent, the inflammatory reaction can promote the development of cancer (12). In addition, inflammation, particularly chronic inflammation that is constantly stimulated, can lead to immune tolerance in the cells that have been wounded, allowing mutant cells to evade immune surveillance and facilitate the invasion of cancer (13). Inflammation is directly linked to the development, invasion, and metastasis of malignancies (14, 15). In recent years, the diagnostic and therapeutic value of inflammatory markers has been studied, which provides us with new research ideas for cancer treatment. Numerous inflammatory indicators, including neutrophil-to-lymphocyte ratio (NLR), platelet-to-lymphocyte ratio (PLR), and system immune-inflammatory index (SII), are highly predictive of cancer (16–20).

The-pan-immune-inflammation value (PIV), a parameter calculated from platelet, neutrophil, monocyte, and lymphocyte counts, is a newly defined inflammation-related index. PIV was initially researched in patients with metastatic colorectal cancer in 2020, and the findings demonstrated that PIV had a substantial prognostic value for patients’ survival as well as that its predictive efficiency was significantly greater than that of previously known inflammation-related markers (21).

The connection between PIV and the prognosis of breast cancer patients has been the subject of some retrospective investigations so far. However, the findings were inconsistent. Sahin et al. found that patients with low PIV had a better prognosis (22). Instead, the results of Truffi et al. showed that PIV was not linked to the prognosis of women suffering from breast cancer (23). A systematic evaluation is currently lacking, and it is still unknown what role PIV has in predicting prognosis in breast cancer. Therefore, we designed the first meta-analysis to investigate whether PIV could be a reliable prognostic biomarker in patients with breast cancer.

## 2 Methods

### 2.1 Literature search strategies

The proposal for this meta-analysis was drafted following the project guidance of the PRISMA statement (24). The PubMed, EMBASE, the Cochrane Library, and the CNKI were searched using the following keywords: “pan-immune-inflammation-value”, “PIV”, “breast neoplasms”, “breast tumor”, and “breast cancer”, in April 2023. For detailed search strategies, see **Table S1**. We also searched the grey literature on Google Scholar. The reference lists of the publications that meet preset criteria are manually retrieved to ensure the comprehensiveness of the data.

### 2.2 Inclusion and exclusion criteria

The pre-established inclusion criteria for this study were as follows: patients with a diagnosis of breast cancer; the prognostic value of PIV was assessed; any of the three outcomes, overall survival (OS), pathological complete response (pCR), and progression-free survival (PFS), was available; The clinical data were complete, and the follow-up data were complete and reliable. Apart from case reports, conference abstracts, and comments. The literature with unpublished endpoints or ambiguous statistics was also removed.

### 2.3 Data extraction and quality assessment

The following data were extracted from the baseline characteristics of the included studies: author, year, study region, study design, study period, sample size, patient age, treatment, cut-off, and outcome measures. To rate the caliber of observational studies, we used the Newcastle-Ottawa Scale (NOS) results. The NOS consists of three parts: selection (0-4 points), comparability (0-2 points), and outcome assessment (0-3 points). Articles with a NOS score of more than 6 were rated as high quality. Xiaoyan Qi and Qi Jin separately judged the risk of bias in the literature and double-checked the results. In the event of disagreement, it will be decided by discussion or by seeking help from a third party.

### 2.4 Statistical methods

All the data included in this meta-analysis were analyzed using Stata 15.0 software. For studies that provided both multivariable analysis and univariate analysis, we gave priority to extracting the former. The chi-square test was utilized to evaluate the heterogeneity between studies. When *P* < 0.1 and I²> 50%, it indicated the presence of heterogeneity and was appropriate to employ a random effects model, whereas the fixed effect model was applied. A sensitivity analysis was performed by removing a single study. Publication bias was assessed using Begg’s and Egger’s tests (25, 26).

## 3 Results

### 3.1 Characteristics of studies

**Figure 1:**
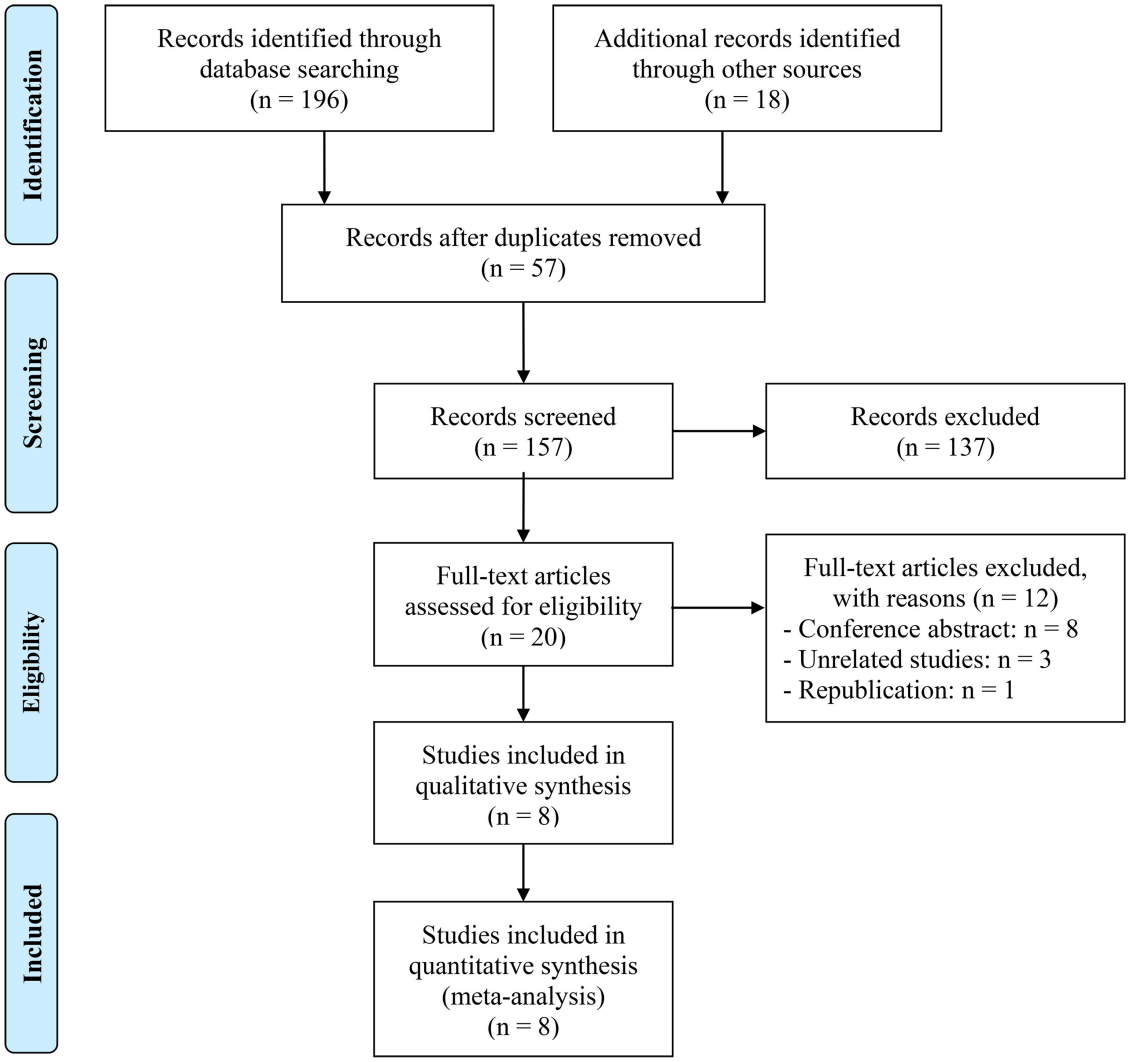
The PRISMA flowchart.

A total of 57 replicate studies were eliminated after an initial search. With a rigorous examination of the abstracts and titles, 137 items were eliminated. The remaining 20 articles’ entire texts were then assessed in further detail. There were 8 articles involving 2953 patients (21, 22, 27–32). **Figure 1** displays the PRISMA flowchart.

**Table 1** lists the basic characteristics of the research that was included. Seven of the nine studies included the outcome OS, five included PFS, and two included pCR. Furthermore, four studies were undertaken in Italy, one in Japan, and two in China and Turkey. According to reports, the cutoff point for PIV was between 205.1 and 438.7. Eight articles earned NOS values ranging from 6 to 8, indicating a low probability of bias.

**Table 1:** Main characteristics of the studies included.

Studies	Study region	Study design	Study period	Sample size	Age (Years)	Treatment	Cut-off	Out comes	NOS
Yamanouchi et al., 2023 (32)	Japan	Retrospective	01/2012-12/2021	35	64 (37-98)^c^	Systemic therapy	205.1	OS	8
Provenzano et al., 2023 (1) (31)	Italy	Retrospective	04/2007-04/2022	78	-	Chemotherapy	NA^d^	OS, PFS	7
Provenzano et al., 2023 (2) (31)	Italy	Retrospective	09/2008-02/2020	96	-	Chemotherapy	NA^d^	OS, PFS	7
Demir et al., 2022 (28)	Turkey	Retrospective	01/2010-12/2020	243	36(21-40)^a^	-	301.0	OS	8
Geng et al,. 2022 (30)	China	Retrospective	01/2019-12/2021	172	50(25-73)^a^	Chemotherapy	-	pCR	6
Lin et al., 2022 (27)	China	Retrospective	12/2010-10/2012	1312	48(41-57)^b^	Surgery	310.2	OS	7
Truffi et al., 2022 (23)	Italy	Retrospective	-	217	52 ± 11^c^	NAC	438.7	PFS	7
Ligorio et al., 2021 (29)	Italy	Retrospective	04/2014-09/2020	57	-	Taxane-trastuzumab-pertuzumab biochemotherapy	285.0	OS, PFS	7
Sahin et al., 2021 (22)	Turkey	Retrospective	01/2008-12/2019	743	48(22-84)^a^	Chemotherapy	306.4	OS, PFS, pCR	8

^a^Median (range); ^b^Median (quartiles); ^c^Means ± standard deviations; pCR, pathological complete response; PFS, progression-free survival; OS, overall survival.

^d^Patients were ranked from lowest to highest according to PIV value, and the PIV value of the 80%th patient was taken as the cutoff value.

### 3.2 PIV and OS

**Figure 2:**
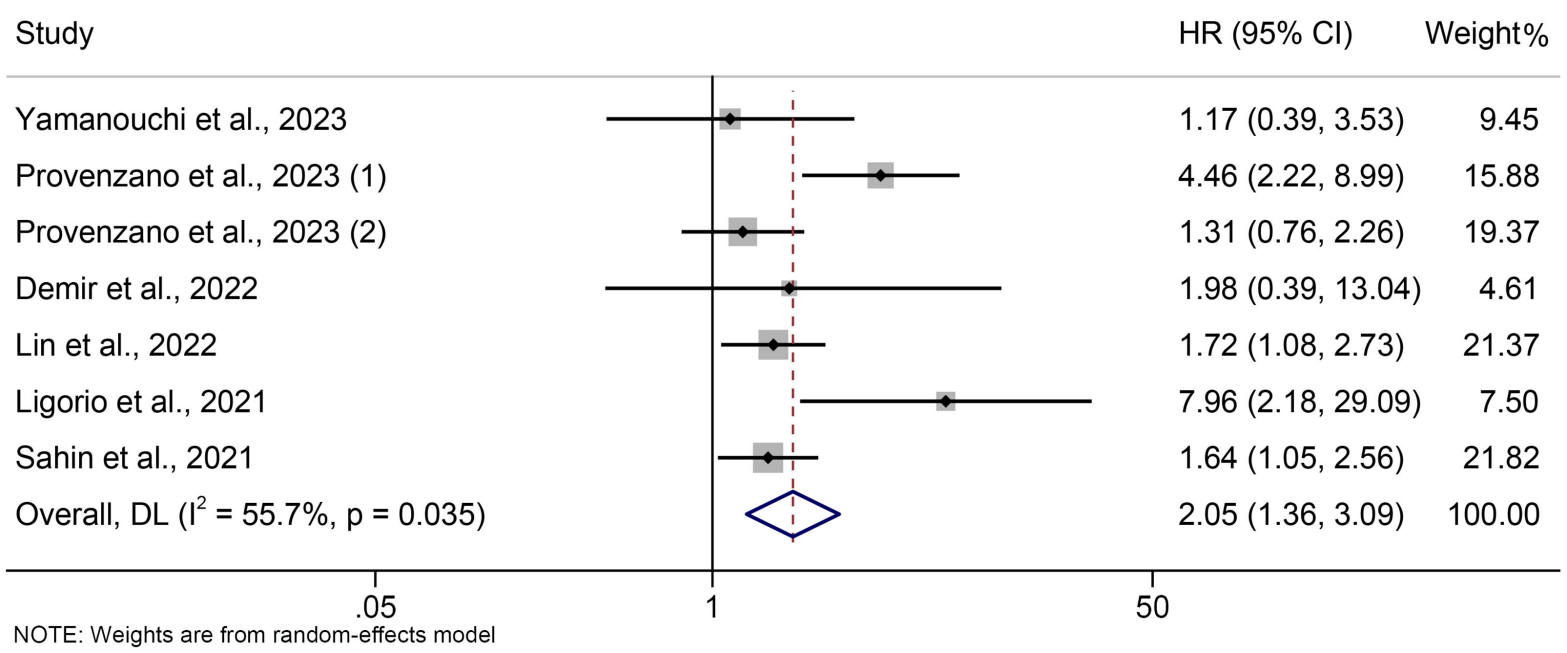
The association between PIV levels and OS. OS, overall survival; HR, hazard ratio; CL, confidence interval; PIV, pan-immune-inflammation value.

The association between PIV and OS was examined using prognostic data from seven studies involving 2564 breast cancer patients. As shown in **Figure 2**, the pooled HR was 2.045 (95% CI: 1.355-3.086, *P* = 0.001), indicating that a high PIV increased the chance of mortality by 104.5%. We discovered significant heterogeneity amongst the included studies, so we used a random effect model (I² = 55.7%, *P* = 0.035).

**Figure 3:**
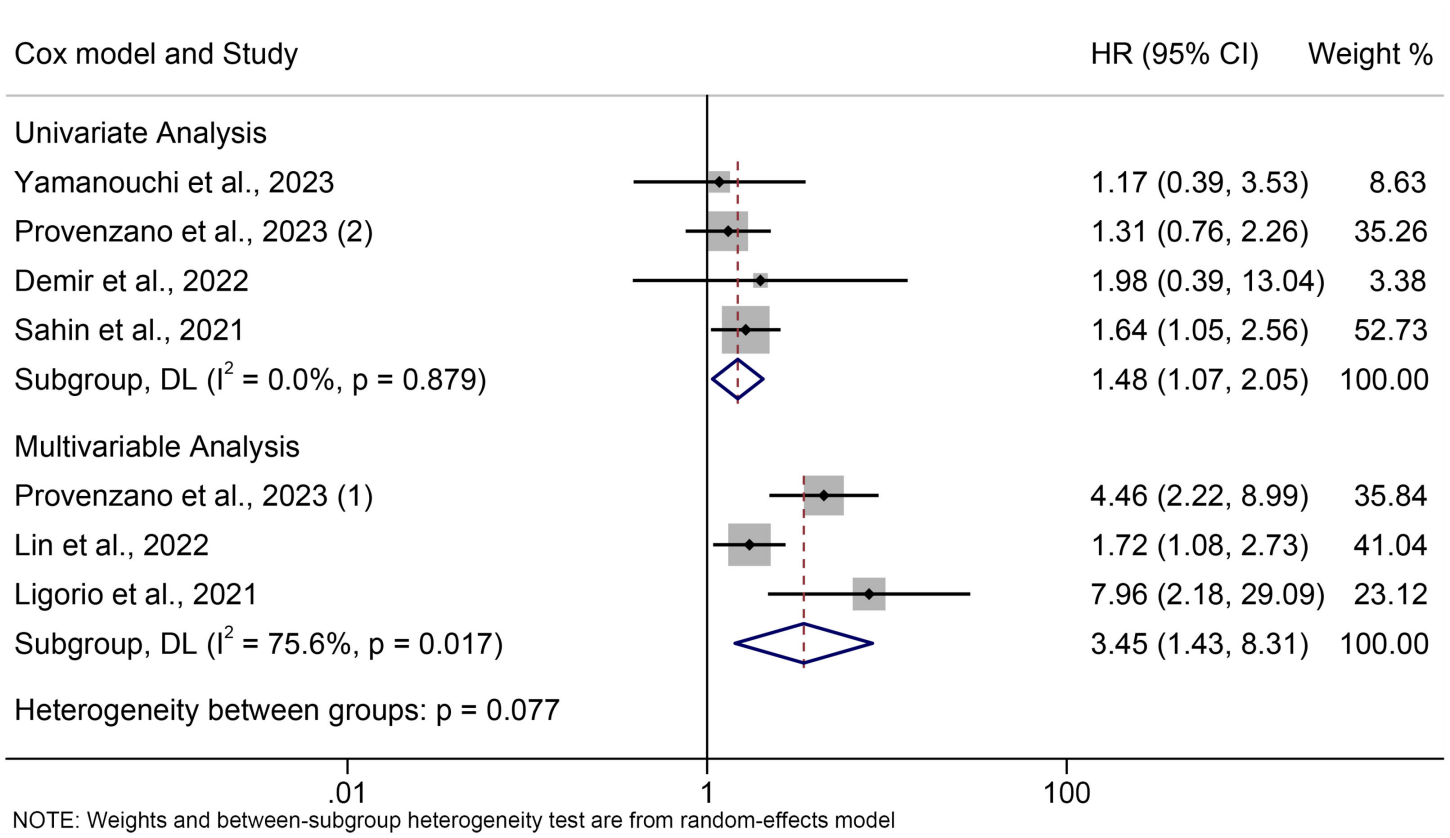
Subgroup analysis of the association between PIV levels and OS. OS, overall survival; HR, hazard ratio; CL, confidence interval; PIV, pan-immune-inflammation value.

The subgroup analyses were carried out in line with the type of analysis. The data demonstrated that the univariate and multivariate meta-results were consistent (**Figure 3**).

### 3.3 PIV and PFS

**Figure 4:**
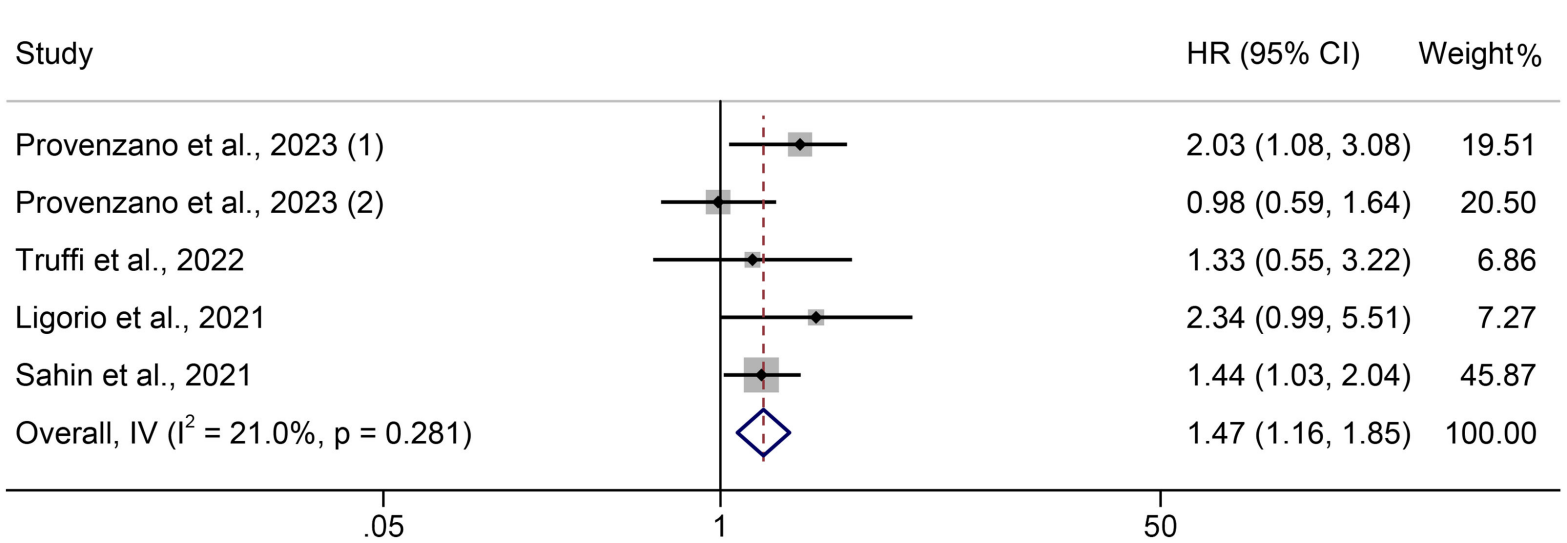
The association between PIV levels and PFS. PFS, progression-free survival; HR, hazard ratio; CL, confidence interval; PIV, pan-immune-inflammation value.

Five trials involving 1191 people were analyzed to identify the relationship between PIV and PFS in breast cancer patients. A fixed effects model was applied since there was no discernible heterogeneity in the included trials (I² =21.0%, *P* = 0.281, **Figure 4**). Compared to persons with low PIV, those with high PIV had a shorter PFS, according to the assessments (HR = 1.466, 95% CI: 1.163-1.848, *P* = 0.001, **Figure 4**).

### 3.4 PIV and pCR

Two of these studies investigated the correlation between PIV and the response to neoadjuvant chemotherapy in breast cancer. In Sahin’s study, PIV was analyzed as a binary variable, while in Geng’s study, PIV was treated as a continuous variable. As a result of the significant heterogeneity between studies, the two studies could not be meta-analyzed. A systematic review was conducted to evaluate whether the evaluation of PIV can predict the pCR to neoadjuvant chemotherapy in breast cancer patients.

Sahin et al. surveyed 743 breast cancer patients in Turkey who underwent NAC and proved that PIV was an independent predictor of NAC response (OR = 3.32; 95% CI: 1.53-7.21, *P* = 0.002). Surprisingly, PIV was found to be superior to other blood-borne inflammation-related indicators in predicting response to NAC in Turkish women with breast cancer for the first time (22). Geng et al. gathered data from 172 breast cancer patients who underwent NAC and completed the operation. Multivariate analysis revealed that a low PIV (OR = 0.996, 95% CI = 0.993-1.000, *P* < 0.05) was an independent favorable factor for neoadjuvant chemotherapy efficacy (30). Both studies confirmed the predictive value of PIV for the response to neoadjuvant therapy in breast cancer.

### 3.5 Sensitivity analysis

**Figure 5:**
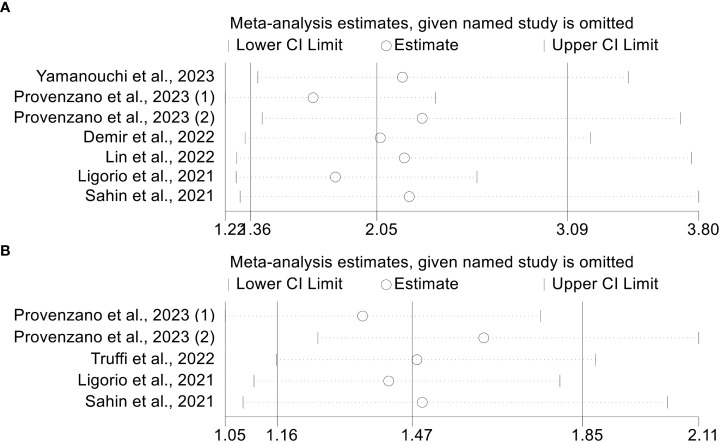
Sensitivity analysis of OS **(A)** and PFS **(B)**. CL, confidence interval

The leave-one-out approach was used to conduct a sensitivity analysis. By omitting one trial at a time, we discovered that the pooled HR for OS did not change significantly, varying from 1.697 (95% CI: 1.218-2.365, after excluding Provenzano et al., 2023(1)) to 2.292 (95% CI: 1.419-3.702, after excluding Provenzano et al., 2023(2), **Figure 5A**). Furthermore, the pooled HR for PFS did not change considerably, ranging from 1.355 (95% CI: 1.047-1.754, after eliminating Provenzano et al., 2023(1)) to 1.626 (95% CI: 1.255-2.108, after omitting Provenzano et al., 2023(2), **Figure 5B**). The above results indicate that our results are robust and credible.

### 3.6 Publication bias

There was no evidence of publication bias for OS (Egger’s test: *P* = 0.364, Begg’s test: *P* = 0.548) or PFS (Egger’s test: *P* = 0.689, Begg’s test: *P* = 1.000). From that, we can see that our outcome was robust.

## 4 Discussion

The purpose of our study was to investigate the prognostic significance of PIV in patients with breast cancer. To the best of our knowledge, the present study is the first meta-analysis to evaluate the association between PIV and breast cancer survival outcomes. In this meta-analysis involving 2953 breast cancer patients, a clear relationship between higher PIV and shorter OS and PFS was observed. Of note, we also found that PIV showed excellent predictive value for NAC efficacy in breast cancer, with low levels of PIV being an independent favorable factor for NAC efficacy. Considering that PIV does not belong to the same variable type in the two included studies, resulting in too much heterogeneity and not being able to guarantee the stability of the results, only a qualitative description is carried out in this meta-analysis, which also needs to be further verified by more follow-up studies.

There is a strong link between inflammation and cancer (33, 34). On the one hand, an inflammatory response can, to a certain extent, lead to the occurrence of cancer (35, 36). On the other hand, cancer that has occurred can also stimulate the development of inflammation because of various genetic and carcinogenic changes, providing an environment for cancer development and further promoting the process of cancer (12). Inflammation can promote the proliferation and survival of malignant tumor cells, promote the generation and metastasis of blood vessels, weaken the body’s immune response, and change the body’s response to hormones and drugs. Therefore, inflammation makes survival shorter, leading to a poor patient prognosis (37–41). Although tumor activity and patient prognosis may be somewhat predicted by inflammatory conditions, the use of conventional tissue-based tumor-related biomarkers in clinical practice is constrained by issues like challenging specimen collection and high cost (42). Complete blood count (CBC)-based biomarkers were first introduced by Roman Zahorec in 2001 to address this limitation, with the advantages of easy availability and low cost (43). Counts of lymphocytes, neutrophils, platelets, and monocytes are all included in a complete blood count. Over the past decade, there has been substantial evidence that CBC-based biomarkers, such as NLR and PLR, have good prognostic value for a variety of cancers (44–47).

Some studies have found that lymphocyte has anti-tumor immune activity, can inhibit tumor cell growth, and stimulate tumor cell apoptosis (48). Neutrophils can secrete tumor growth factors, which can accelerate the proliferation and migration of tumor cells, and inhibit the anti-tumor activity of lymphocytes to a certain extent (49). Monocytes can differentiate into tumor-associated macrophages, and most of the macrophages can produce angiogenic factors to promote the formation of tumor neovascularization (50). In addition, proteases secreted by monocytes degrade the extracellular matrix to a certain extent. Platelets can enhance the defense role of tumor cells against immune cells, secrete growth factors, and release activators, thus accelerating the proliferation of tumor cells (51). PIV is a newly defined inflammation-related index. It is calculated as neutrophil count (10^9^/L) × monocyte count (10^9^/L) × platelet count (10^9^/L)/lymphocyte count (10^9^/L). As mentioned above, different blood cell populations reflect and regulate different aspects of anti-tumor resistance. In contrast to earlier indices (NLR and PLR), PIV combines all routinely assessed blood cell populations that reflect systemic inflammation and immunity. As a result, it provides a more complete picture of the host’s condition and may have a more reliable prognostic value for cancer. PIV has been shown by Ligorio et al. to be a unique and useful predictor of OS in advanced BC patients with HER-2 positivity who were given first-line trastuzumab-pertuzumab biochemotherapy (29). Sahin et al. also found that PIV had better predictive power than NLR, PLR, monocyte-to-lymphocyte ratio (MLR), and systemic immune inflammation index (SII) in breast cancer patients (22). More surprisingly, Lin et al. observed that PIV predicted OS with higher accuracy than traditional TNM staging systems (27). Therefore, compared with other biomarkers, PIV may have better prognostic value and clinical utility in breast cancer patients. In conclusion, our study finds that PIV can be a useful tool for predicting poor outcomes in breast cancer patients. Individualized and timely immunization interventions may improve outcomes in patients with high baseline PIV.

Our analysis still has certain limitations. The number of patients included in this study was relatively limited. It was also not possible to further evaluate the correlation between PIV and the prognosis of patients with different molecular subtypes of breast cancer. This meta-analysis included studies that only used PIV values at a single time point and lacked dynamic assessment, which was an important limitation. The included studies were heterogeneous in terms of tumor type and treatment type, reducing the generalizability of the results. In addition, there is a very important point: because PIV has different reference values in studies conducted in different populations around the world, there is no uniform cut-off value, which may cause the final summary results to be different from the actual value. Therefore, more high-quality studies with large sample sizes, especially multicenter RCTs, are needed to verify and refresh our conclusions. And these studies should include patients from different regions and ethnicities, and discuss uniform cutoff values to open the way for the clinical application of this biomarker.

## Data availability statement

The original contributions presented in the study are included in the article/**Supplementary Material**. Further inquiries can be directed to the corresponding author.

## Author contributions

XQ, BQ, and DL conceived and designed the study. XQ, BQ, TS, DH, HZ, YL, QJ, and MY were responsible for the collection and assembly of data, data analysis, and interpretation. XQ and BQ were involved in writing the manuscript. XQ, BQ, and DL revised the manuscript. All the work was performed under DL instructions. All authors contributed to the article and approved the submitted version.

## Supplementary material

Supplementary material 1 The detailed search strategies for Pubmed.
