# Supplementary material for: Clinical utility of the pan-immune-inflammation value in breast cancer patients
Source: Front Oncol. 2023 Aug 30;13:1223786. doi: 10.3389/fonc.2023.1223786 (PMC10499041; doi:10.3389/fonc.2023.1223786)
Supplement: Supplementary material 1 — The detailed search strategies for Pubmed. [file DataSheet_1.docx]

Retrieval strategies in Pubmed

(((((pan-immune-inflammation-value) OR (pan-immune-inflammation value)) OR (pan immune inflammation value)) OR (PIV))) AND ((((((((((((((((((((((((((((((Breast Neoplasm[Title/Abstract]) OR (Neoplasm, Breast[Title/Abstract])) OR (Breast Tumors[Title/Abstract])) OR (Breast Tumor[Title/Abstract])) OR (Tumor, Breast[Title/Abstract])) OR (Tumors, Breast[Title/Abstract])) OR (Neoplasms, Breast[Title/Abstract])) OR (Breast Cancer[Title/Abstract])) OR (Cancer, Breast[Title/Abstract])) OR (Mammary Cancer[Title/Abstract])) OR (Cancer, Mammary[Title/Abstract])) OR (Cancers, Mammary[Title/Abstract])) OR (Mammary Cancers[Title/Abstract])) OR (Malignant Neoplasm of Breast[Title/Abstract])) OR (Breast Malignant Neoplasm[Title/Abstract])) OR (Breast Malignant Neoplasms[Title/Abstract])) OR (Malignant Tumor of Breast[Title/Abstract])) OR (Breast Malignant Tumor[Title/Abstract])) OR (Breast Malignant Tumors[Title/Abstract])) OR (Cancer of Breast[Title/Abstract])) OR (Cancer of the Breast[Title/Abstract])) OR (Mammary Carcinoma[Title/Abstract])) OR (Mammary Carcinomas[Title/Abstract])) OR (Breast Carcinoma[Title/Abstract])) OR (Breast Carcinomas[Title/Abstract])) OR (Carcinoma, Breast[Title/Abstract])) OR (Carcinomas, Breast[Title/Abstract])))) OR ("Breast Neoplasms"[Mesh]))
